# *Hibiscus sabdariffa* in Diabetes Prevention and Treatment—Does It Work? An Evidence-Based Review

**DOI:** 10.3390/foods11142134

**Published:** 2022-07-19

**Authors:** Daniel Jamrozik, Weronika Borymska, Ilona Kaczmarczyk-Żebrowska

**Affiliations:** Department of Pharmacognosy and Phytochemistry, Faculty of Pharmaceutical Sciences in Sosnowiec, Medical University of Silesia, Katowice, Jagiellońska 4, 41-200 Sosnowiec, Poland; weronika.borymska@sum.edu.pl (W.B.); izebrowska@sum.edu.pl (I.K.-Ż.)

**Keywords:** diabetes, *Hibiscus sabdariffa*, extracts, hypoglycemic, antioxidant, antilipidemic, in vitro, animals, patients

## Abstract

Diabetes is currently a global health problem that is already reported as an epidemic. This metabolic disease, characterized by a disturbance in the carbohydrate, protein, and lipid metabolism, is often accompanied by disorders of several organs. Its treatment is expensive and often difficult to control. Therefore, it seems necessary to search for new drugs and solutions to facilitate therapy and reduce treatment costs. Herbal medicines are becoming more and more popular. *Hibiscus sabdariffa* (roselle) is a plant that grows wild in a tropical climate. It has been used in folk medicine for thousands of years. Thanks to the numerous active compounds, including polyphenols, polysaccharides, organic acids, or pectins, it is reported to exhibit hypoglycemic, antioxidant, hypotensive, and anti-lipidemic activities and numerous indirect effects that are related to them. The aim of this review was to update the knowledge about the therapeutic effects of roselle in diabetes and its comorbidities based on in vitro, animal, and human studies. After a careful analysis of the scientific literature, it can be stated that roselle is a promising product that can be used either on its own or as an addition to the conventional treatment regimens to prevent or treat diabetes and its accompanying diseases.

## 1. *Hibiscus sabdariffa*—Characteristic

*Hibiscus sabdariffa* (roselle) is a plant belonging to the Malvaceae family, growing wild in tropical climates [1]. Due to the high content of pharmacologically active compounds and thus good healing properties, roselle has been well known and widely used in traditional medicine for thousands of years [2,3]. In addition to its medical use, roselle is widely used in the culinary arts as a source of nutrients or as a natural dye that is used in the food and textile industries [4,5,6]. Today it is cultivated almost everywhere in the tropics and subtropics; in Mexico, India, Saudi Arabia, China, Vietnam, Sudan, Egypt, Malaysia, Indonesia, the Philippines, and Nigeria [7]. It is known by many names depending on the geographical area, for example Karkadé, Roselle, Jamaica, Sorrel, and many others [3].

### 1.1. Morphology

Roselle belongs to a genus of about 300 species. These are annual or perennial herbs, semi-shrubs, and shrubs. They can grow up to 2.5 m in height. The stem is smooth or almost smooth, cylindrical, and red in color. The leaves are up to 15 cm in size, arranged alternately, deeply lobed, with 3–5 or even seven parts. The leaf edge is toothed. The flowers are 8–12 cm wide and occur singly in the leaf axils or terminal. They are white, pale yellow, yellow, rose, or with a maroon eye. The calyx has five large sepals with a collar (epicalyx) of 8 to 12 thin, pointed bracts (or bracteoles) around the base. The calyx is 2 cm wide and about 6 cm long, and it becomes fleshy and red as the fruit matures. Maturing takes about six months. After this time, the capsule breaks and the seeds spill out. The seeds are kidney-shaped, light brown, and up to 5 mm long [2,7,8]. The flower calyces are usually red (rose), but there are also white and purple varieties [8,9].

### 1.2. Roselle as a Functional Food and Nutraceutical

Roselle is widely used as a source of nutrients and natural dyes in the food industry [4,5]. Many parts of roselle can be consumed, but the calyces are the most popular in culinary products [10]. The calyces are used in the production of juices and soft drinks. Fresh or dried, they are also used in the production of ice cream, jams, wine, chocolate, and fermented beverages or as an additive to improve the taste of other beverages. The calyces can also be pickled [4,5,10,11,12]. Aqueous preparations such as decoction or infusions are generally regarded as safe and can be consumed as hot or cold drinks. These low-calorie drinks possess little carbohydrates and are characterized by a high acid content, thus are sour in taste. To soothe this taste, many additives such as fruits or sweeteners may be mixed with roselle-containing drinks [5,10]. The reports regarding the nutritional value of the roselle calyces differ. These variations result from many factors, including the genotype of the plant, its color or cultivar, as well as its origin [4,10]. Apart from being a source of dietary fiber, protein, carbohydrates, and small amounts of lipids, the roselle calyces are also a source of other nutrients. There are vitamins such as riboflavin, thiamine or niacin, β-carotene as a provitamin A, and a high content of ascorbic acid [5,10,12,13]. The calyces are also a good source of macro- and micro-elements, including iron, calcium, phosphorus, magnesium, sodium, or zinc [10,12,13,14]. Additionally, *Hibiscus sabdariffa* is also a great source of phytochemicals, which will be described in the next paragraph in more detail.

Since roselle is such an abundant source of active components, numerous publications indicate that it can be useful in the treatment or prevention of many chronic diseases [4,7,13]. The foods rich in bioactive compounds which are able, apart from their nutritional function, to improve the state of the human body, and prevent or even treat chronic diseases are called functional foods or nutraceuticals [15,16]. Therefore, roselle may be called functional food [17] as it is a good candidate for nutraceutical purposes [1].

### 1.3. Hibiscus sabdariffa Active Compounds

Roselle calyces owe their therapeutic effects to numerous pharmacologically active compounds [5]. The white and rose roselle variants differ in the content of active compounds, but only to a slight extent; the red variety is characterized by a higher content of total phenols, flavonoids, and anthocyanins than the white variety. The white variety is characterized by a higher ability to scavenge free radicals than the red variety [8]. Another study demonstrated that purple calyces exhibit stronger antioxidative activity and contain more flavonoids than red ones [9]. However, most studies are conducted on the active compounds of the red variety. Numerous publications indicate that roselle calyces and other parts of the plant are rich in polyphenols (anthocyanins, flavonoids, phenolic acids, tannins), polysaccharides, pectins, non-phenolic organic acids, and carotenoids [4,7,18,19,20,21]. The most important organic acids in roselle are hibiscus acid, hydroxycitric acid, malic acid, ascorbic acid, oxalic acid, succinic acid, tartaric acid, arachidic acid, and citric acid [2,7,18,22]. Roselle owes its antioxidant effect to polyphenols [2,21]. The most important anthocyanins in roselle are delphinidin-3-sambubioside and cyanidin-3-sambubioside [2,7,18]. Of the flavonoids that have been isolated from roselle, the most important are quercetin, hibiscetin (hibiscetin-3-glucoside), sabdaritrin, gossypitrin, and other gossypetin glycosides, and luteolin. There are also phenolic acids such as chlorogenic acid, protocatechuic acid, and caffeic acid, and many other phytochemicals [2,7,18,23]. Sugars in the roselle flower petals are mainly galactose, galacturonic acid, rhamnose, arabinose, glucose, mannose, and xylose [7,23]. Some authors also indicate that in the calyces there are triterpenoids such as α-amyrin and lupeol [24]. Roselle calyces are also rich in calcium, magnesium, iron, trace elements, and vitamins [4,5]. It may also be important that there are reports that the content of roselle active ingredients depends on seasonal variations in the crops (dry or wet planting seasons). The research results indicate that roselle planted in the dry season contains significantly more anthocyanins, fructose, glucose, and malic acid than those planted during the wet season. On the other hand, the citric acid content was lower in the dry season than in the wet season. Therefore, these results make it possible to manipulate the yields to obtain plants with the desired composition of active compounds [25]. It is also interesting that there are compounds that affect the stability of polyphenols and anthocyanins of roselle. One of them is stevia. By demonstrating the ability to inhibit the activity of α-amylase and reduce the degradation of polyphenols, including anthocyanins, it extends the storage time of roselle drinks. As such, it can be a safe and suitable ingredient in diabetic drinks based on roselle extracts [26]. Roselle active compounds have been collected and listed in Table 1.

### 1.4. Medicinal Properties and Usage of Roselle

Roselle is a plant valued worldwide because all of its parts (seeds, stems, flowers, leaves) have medicinal, industrial, or other uses [2]. From the medicinal point of view, the most valuable part of roselle are the flower calyces [27]. Already in ancient China, its medicinal value was appreciated, and tea with roselle was used to treat hypertension, fever, kidney and bladder stones, and inflammation [8,28]. Roselle flowers and seeds are also used in the ethnomedicine of South American peoples. They cleanse the blood and liver and reduce hypertension [29]. In India, Mexico, and Africa, roselle leaves and calyx infusions were made. They were choleretic, diuretic, antipyretic, and hypotensive, and improved intestinal peristalsis and blood viscosity [7]. In India, it is used in order to relieve pain in urination and indigestion, and in Thailand to treat stones in the kidneys and urinary bladder [2]. In Egypt, infusions of roselle were used to treat heart disease, neuropathies, and lower body temperature [7]. The literature also indicates that roselle has antidiabetic, hypolipidemic, hepatoprotective, neuroprotective, antihypertensive, and vasodilating effects. It also acts as an anti-inflammatory agent by reducing the levels of tumor necrosis factor α (TNF-α). Numerous reviews also confirm the beneficial use of roselle in treating metabolic syndrome and tumor metastasis and as an antiseptic, emollient, and laxative. Roselle owes its popularity to its ability to improve the level of lipids, blood glucose, and blood pressure [4,5,18,23,30,31,32,33,34,35].

## 2. Diabetes Mellitus

Diabetes mellitus (DM) is a chronic metabolic disease characterized by elevated blood glucose levels. Over time, it can seriously damage the heart, blood vessels, eyes, kidneys, and nerves [36]. There are several types of diabetes, but two of them are dominant: type 1 diabetes mellitus (T1DM) and type 2 diabetes mellitus (T2DM) [36]. T1DM is generally revealed in adolescence and is one of the most common chronic diseases in childhood [37]. It is caused by the destruction of the β-cells of the Langerhans islets in the pancreas by the immune system (autoimmune process). As a result, the person’s body produces very little or no insulin [38]. Patients with this type of diabetes (approximately 5% of all diabetes cases) are treated with various types of exogenous insulin (short-, medium-, and long-acting insulin) because without insulin, they would not be able to survive [39,40,41]. Thanks to this approach, the survival rate of patients with T1DM has increased over the past 30 years [37]. T2DM is characterized by decreased insulin sensitivity of tissues (especially skeletal muscles and liver) with concomitant insulin resistance and impaired insulin secretion by the β-cells of the Langerhans islets in the pancreas [42,43]. It accounts for over 90% of all cases among diabetic patients [42,43]. The most effective method of its treatment is taking oral antidiabetic drugs with multidirectional pharmacological action. Often monotherapy is insufficient, and it is necessary to use several antidiabetic drugs simultaneously [44,45]. T2DM may be an outcome of metabolic syndrome—a set of risk factors such as central obesity, hypertension, insulin resistance, or dyslipidemia [46].

Diabetes is not only about elevated blood glucose levels. It is a disease that is accompanied by numerous other disorders. A diabetic person may struggle with hypercholesterolemia, dyslipidemia, impaired protein metabolism, diabetic retinopathy, nephropathies, neuropathies, ulcerations, and inflammations [38,47,48].

In diabetes, there is an increased production of reactive oxygen species (ROS), which results in the formation of oxidative stress. Reactive oxygen species cause the oxidation of cellular components. It is the leading cause of the above-mentioned complications and diseases accompanying diabetic disease [38,49].

Diabetes treatment is very expensive; therefore, it becomes useful to search for new solutions that can replace, supplement, or support the action of synthetic antidiabetic drugs. There are many reports on the effectiveness of herbal drugs that are used for this disease [50]. Roselle has gained a high popularity among patients suffering from diabetes, and their belief is supported by in vitro, and in vivo tests [51,52]; therefore, this publication is devoted to this plant. The aim of the work is a review of the literature describing the use of roselle in the treatment of diabetes mellitus and its accompanying diseases. Roselle has been the subject of many original and reviewed papers, including conditions that may be associated with diabetes and metabolic syndrome [35,53,54,55,56,57,58,59,60,61,62,63,64,65,66,67,68]. However, the existing works are older or incompletely related to diabetes, and research progress on this topic is very large [69,70,71,72]. Therefore, there is a need to update the current knowledge. The following medical databases were searched for the study: PubMed, Embase, Google Schoolar, Cochrane, and UpToDate. Articles were searched for by entering the following keywords: “*Hibiscus sabdariffa*”, “diabetes”, “hypoglycemic”, and “hyperglycemia”. Duplicates, works written in a language other than English, reading abstracts, and non-relevant papers were excluded from this review. The time scope of the articles was not set during the search. The oldest work used in this review is from 2000 and the newest works are from 2022.

## 3. *Hibiscus sabdariffa* and Its Therapeutic Effects Studied In Vitro

**Figure 1:**
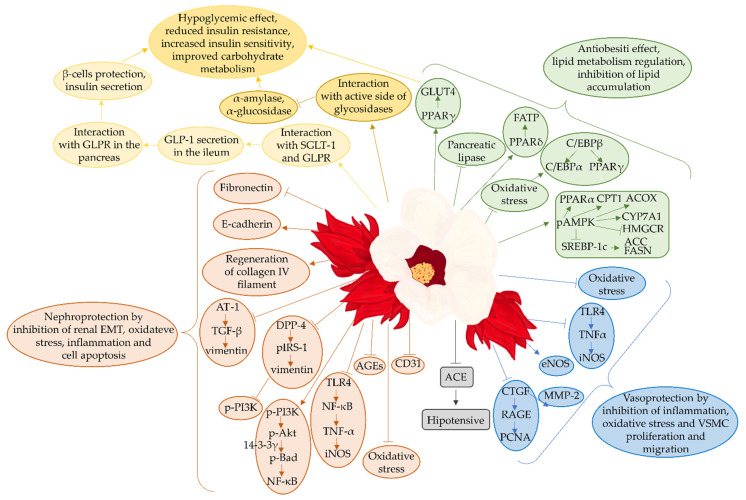
Molecular mechanisms of roselle action in multiple diabetes-related conditions. Mechanisms described in oval shapes were examined in vitro or in vivo in diabetic models/conditions, while those presented in rectangular boxes were explored in other models/conditions but are putative also for diabetes. ACC—acetyl-CoA Carboxylase; ACE—angiotensin converting enzyme; ACOX—peroxisomal acyl-coenzyme A oxidase; AGEs—advanced glycation end-products; p-Akt—phospho-protein kinase B; pAMPK—phospho-AMP-activated protein kinase; AT-1—angiotensin II receptor; p-Bad—phospho-BCL2-associated agonist of cell death; CD31—cluster of differentiation 31; C/EBPα—CATT/enhancer binding protein-α; C/EBPβ—CATT/enhancer binding protein-β; CPT1—carnitine palmitoyltransferase1; CTGF—connective tissue growth factor; CYP7A1—cytochrome P450 7A1; DPP-4—type 4 dipeptidyl peptidase; EMT—mesenchymal epithelial transition; eNOS—endothelial NO synthase; FASN—fatty acid synthase; FATP—fatty acid transporter protein; GLP-1—glucagon-like peptide 1; GLPR—glucagon-like peptide receptor; GLUT4—glucose transporter type 4; HMGCR—3-hydroxy-3-methylglutaryl-CoA reductase; iNOS—inducible NO synthase; pIRS-1—phospho-insulin receptor substrate 1; MMP-2—migration–matrix metalloproteinase 2; NF-κB—nuclear factor kappa-light-chain-enhancer of activated B cells; PCNA—proliferating cell nuclear antigen; p-PI3K—phospho-phosphatidylinositol 3-kinase; PPARα—proximal proliferator-activated receptor-α; PPARγ—proximal proliferator-activated receptor-γ; PPARδ—proximal proliferator-activated receptor-δ; RAGE—receptor of advanced glycation end product; SGLT-1—sodium-glucose co-transporter-1; SREBP-1c—sterol regulatory element-binding protein-1 c; TGF-β—transforming growth factor β1; TLR4—toll-like receptor 4; TNF-α—tumor necrosis factor α; VSMC—vascular smooth muscle cells.

In treating diabetes mellitus, it is crucial to control postprandial hyperglycemia. It is important in the initial stage of disease treatment because it may reduce the likelihood of future complications. In practice, the use of inhibitors of carbohydrate hydrolyzing enzymes, intestinal α-glucosidase, and pancreatic α-amylase may contribute to reducing the level of postprandial hyperglycemia. Their action is based on the inhibition of the functions of these enzymes, thus delaying the digestion of complex sugars into simple sugars, reducing the absorption of the latter, and lowering the total blood glucose level [73,74,75,76]. Examples of synthetic inhibitors include orlistat, acarbose, voglibose, and miglitol [77]. However, they have side effects such as abdominal gas and diarrhea. It may be caused by the excessive inhibition of the pancreatic α-amylase function and thus the excessive fermentation of sugars accumulated in the intestine [78]. However, there are publications indicating that plants rich in polyphenolic compounds may also contribute to the inhibition of α-glucosidase and α-amylase, but with a reduced likelihood of side effects [73,74,78]. One of the plants described in the publications with such activity is roselle and its extracts (Figure 1) [31,68,79]. In the study performed by Ademiluyi et al. [33], it was demonstrated that aqueous extracts from red and white roselle calyces revealed weaker inhibitory activity towards α-amylase and α-glucosidase than the reference acarbose drug. Interestingly, red roselle extracts were more potent inhibitors of α-glucosidase (IC_50_ = 25.2 µg/mL) than the white roselle variety (IC_50_ = 47.4 µg/mL), while in the case of α-amylase, the white roselle calyces were stronger inhibitors (IC_50_ = 90.5 µg/mL) than red (IC_50_ = 187.9 µg/mL) [33]. On the other hand, Adisakwattana et al. [73] showed in their research that aqueous extracts (infusions) of roselle flowers, rich in phenolic compounds, anthocyanins, and hibiscus acid, were potent inhibitors of pancreatic α-amylase (IC_50_ = 3.52 mg/mL) and weak inhibitors of intestinal α-glucosidase (IC_50_ > 5 mg/mL). Nevertheless, the authors did not state which variety of roselle they used in the study [73]. The ability to inhibit α-glucosidase may result from the temperature of the aqueous preparation made of the roselle calyces, as demonstrated by Rasheed et al. [80]. It has been proven that cold macerates from roselle calyces show a greater ability to inhibit the action of α-glucosidase compared to hot preparations. The authors also noted that the roselle cultivar is also important, and the α-glucosidase inhibitory effect correlates with active components obtained during the making of these preparations. The IC_50_ for cold-prepared extracts was, respectively, IC_50_ = 627 μg/mL for the Aswan variant and IC_50_ = 663 μg/mL for the Sudan variant. For hot-prepared extracts, the results were as follows: IC_50_ = 723 μg/mL for the Aswan variety and IC_50_ = 902 μg/mL for the Sudan variety [80]. The inhibitory effect of aqueous extract from roselle calyx and its fractions on intestinal α-glucosidase and pancreatic α-amylase was also shown by Alegbe et al. [31]. The authors showed that the ethyl acetate fraction of the aqueous extract was the most potent in the inhibition of both these enzymes (the IC_50_ values for α-amylase and α-glucosidase were 411.73 and 433.93 μg/mL, respectively), and further analyses revealed that the compounds responsible for this action were gallic acid and protocatechuic acid [31]. There was also research in which the ability to inhibit intestinal α-glucosidase and pancreatic α-amylase by roselle flower extracts prepared with different solvents was investigated. The roselle flower macerates were made with various solvents: n-hexane, dichloromethane, chloroform, ethyl acetate, and methanol [78]. Each roselle flower extract showed the ability to inhibit α-amylase and α-glucosidase, and in each case, the inhibition of α-amylase was lower than the inhibition of α-glucosidase. Methanolic roselle flower extract showed the strongest ability to inhibit the activity of α-amylase and α-glucosidase, IC_50_ = 50.5 ± 2.0 µg/mL and IC_50_ = 0.7 ± 0.4 µg/mL, respectively. Ethyl acetate extract showed the lowest ability to inhibit α-amylase activity (the IC_50_ was close to 140 µg/mL) and the lowest ability to inhibit α-glucosidase activity (the IC_50_ was about to 50 µg/mL) [78]. In another study conducted on organic fractions obtained from aqueous extract it was shown that the ethyl acetate fraction possessed the highest inhibitory activity towards of both α-amylase and α-glucosidase. This fraction was separated using column chromatography and after nuclear magnetic resonance (NMR) analysis, two components were recognized: gallic acid and protocatechuic acid. Molecular protein-ligand docking revealed that these two compounds, due to their chemical structure, are able to interact with the residues present in the active sides leading to their inhibition [31]. The ethanolic extract prepared from roselle was also demonstrated to inhibit the activity of α-amylase and α-glucosidase, yet it was less potent than acarbose [81]. Also, organic acids, such as hibiscus acid and its 6-methyl ester, which were isolated from methanolic and acetone extracts, respectively, were proven to inhibit α-amylase, but to a lower extent than the positive control [79].

**Figure 2:**
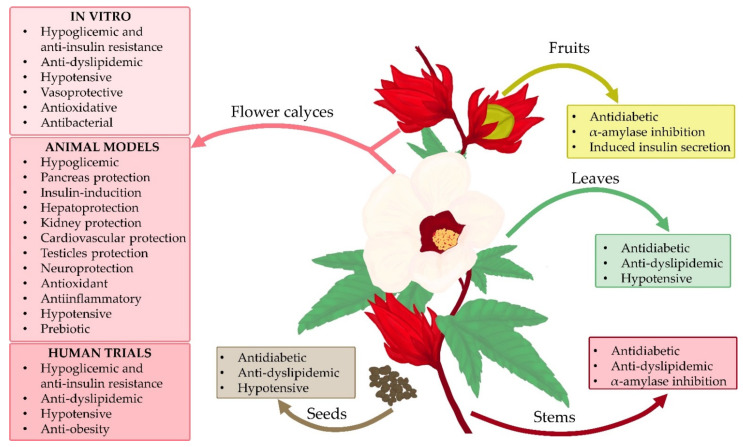
Beneficial effects of different parts of roselle in diabetes and its complications.

Obesity may be the cause of the development of diabetes, especially T2DM [82,83]. Therefore, obesity prevention or treatment can be a good measure to counteract T2DM development. One of the strategies for obesity treatment is to inhibit the activity of pancreatic lipase—an enzyme responsible for breaking down the lipids from food in order to make them available to the body [82]. Buchholz et al. [77] showed in their research that methanolic extracts and aqueous extracts from roselle flowers are able to, to a similar degree, inhibit the activity of pancreatic lipase that is responsible for fat digestion, which may suggest that roselle may reduce the risk of overweight and/or obesity and the likelihood of developing diabetes as a complication of obesity [77]. In addition, researchers have proven that aqueous and methanolic extracts are able, in a similar way, to inhibit the activity of α-amylase, which may contribute to reducing the likelihood of diabetes by indirectly inhibiting the digestion and absorption of sugars. IC_50_ results for lipase were as follows: 0.036 ± 0.001 mg/mL (methanolic extract) and 0.041 ± 0.006 mg/mL (aqueous extract). The IC_50_ results for α-amylase were as follows: 0.029 ± 0.001 mg/mL (methanolic extract) and 0.045 ± 0.001 mg/mL (aqueous extract) [77]. Similar conclusions were reached in another study, in which ethanolic extracts and aqueous extracts from *Hibiscus sabdariffa* flowers were examined. The research revealed that both these extracts were able to inhibit the activity of pancreatic lipase and α-amylase, with ethanolic extracts having a slightly lower capacity to inhibit both these enzymes compared to aqueous extracts. IC_50_ results for lipase were as follows: 151.2 ± 15.17 µg/mL (ethanolic extract) and 108.2 ± 5701 µg/mL (aqueous extract). On the other hand, the IC_50_ results for α-amylase were as follows: 40.22 ± 2898 μg/mL (ethanolic extract) and 35.81 ± 3660 μg/mL (aqueous extract) [84]. Krishnamurthy et al. [85] looked at the effects of aqueous and 50% ethanolic roselle flower extracts on the ability to inhibit pancreatic lipase and intestinal α-glucosidase [85]. They proved that both extracts are inhibitors of the studied enzymes. Pancreatic lipase was more strongly inhibited by aqueous roselle flower extract (IC_50_ = 167.51 ± 2.7 µg/mL). For comparison, the IC_50_ value for a 50% ethanolic extract was 790.65 ± 16.02 µg/mL. Aqueous roselle flower extract was a weaker α-glucosidase inhibitor (IC_50_ = 949.88 ± 10.83 µg/mL) compared to 50% ethanolic extract (IC_50_ = 378.33 ± 4.20 µg/mL) [77]. Another approach to obesity management is the regulation of the proximal proliferator-activated receptor-γ (PPARγ) expression. PPARγ together with proximal proliferator-activated receptor-δ (PPARδ) are key elements in the adipocytes, improving insulin sensitivity. Their simultaneous activation is also crucial to maintaining the lipid/carbohydrate metabolism balance, which was proven in the 3T3-L1 cell line [24]. The dichloromethane extract form roselle calyces at a concentration of 10 μg/mL was added to the differentiated 3T3-L1 adipocytes. As a result, the expression of PPARγ and PPARδ increased in these adipocytes as well as their downstream genes such as fatty acid transporter protein (FATP) and glucose transporter type 4 (GLUT4). This suggests that this type of roselle extract may act as a PPARγ and PPARδ agonist and thus regulate the lipid metabolism and enhance insulin sensitivity. From the compounds which were isolated from this extract, two were identified, based on molecular docking studies, as potential PPARγ agonists—α-amyrin and lupeol [24]. On the other hand, PPARγ is also involved in adipocyte differentiation and is a key element in adipogenesis [86,87]. Therefore, a new approach has been proposed in the treatment and prevention of obesity and diabetes—repressing the PPARγ activity by using its antagonists [88]. Downregulation of the adipogenic gene expression and the prevention of lipid accumulation by suppressing differentiation of 3T3-L1 adipocyte in oxidative stress conditions by roselle aqueous extract was proven. The study revealed that the application of such an extract at concentrations of 0.1, 0.5, and 1.0 mg/mL to the H_2_O_2_-treated 3T3-L1 cells caused a significant reduction of ROS generation. The highest concentration also reduced the elevated by H_2_O_2_ expression of CATT/enhancer binding protein-β (C/EBPβ), which activates the expression of PPARγ, and CATT/enhancer binding protein-α (C/EBPα), which are responsible for adipocyte differentiation and maturation. Moreover, the oil red O staining method used in these adipocytes revealed that H_2_O_2_ induced lipid accumulation in the cells, while roselle treatment suppressed this process. Therefore, roselle aqueous extracts may prevent adipose tissue differentiation or even hypertrophy [89]. In the study performed on human hepatocyte HepG2 cells, which were treated with roselle aqueous extracts (HSE) and polyphenolic fractions from roselle (HPE), concentrations of 0.05 mg/mL, 0.1 mg/mL, and 0.5 mg/mL were used. It was shown that HPE, containing 74% polyphenols, mainly protocatechuic acid, caffeic acid, and gallocatechin gallates, lowered the lipid content in the hepatocytes and regulated 5’AMP-activated protein kinase (AMPK)-dependent processes related to lipids synthesis and metabolism via AMPK phosphorylation. It upregulated the expression of peroxisome proliferator-activated receptor alpha (PPARα), and downregulated the expression of fatty acid synthase (FAS or FASN), HMG-CoA reductase (HMGCoARed or HMGCR), and sterol regulatory element-binding protein-1 c (SREBP-1c) [90]. This HepG2 cell line, but with steatosis induced with oleic acid, was used in order to find whether one of the anthocyanin extracted from roselle can be used as an antisteatotic agent. Delphinidin-3-sambubioside was used at concentrations of 100 and 200 µg/mL and reduced lipid accumulation in the hepatocytes and regulated the genes involved in pathways related to this process. After delphinidin-3-sambubioside treatment, the expression levels of PPARα and carnitine palmitoyltransferase1 (CPT1), which regulate β-oxidation of fatty acids, increased, while the expression of FASN and its upstream regulator SREBP-1c, which are responsible for lipogenesis, decreased (Figure 1) [91]. These results suggest that roselle may be a source of antilipidemic compounds (Figure 2).

Diabetes mellitus, and more precisely the hyperglycemia that is associated with it, together with hypertension, are factors contributing to the development of diabetic nephropathy [92]. Yang et al. 2013 [93], Peng et al. [94], and Huang et al. [95] showed in their research that polyphenolic roselle calyx extracts (HPE) could be an adjuvant preventing the occurrence of diabetic kidney damage. All these experiments were conducted on the HK-2 cell line. However, the cultivation method, incubation time, and type of medium were different [93,94,95]. Yang et al. [93] suggested that the mechanism of HPE nephroprotective action is the regulation of the pathway connected to the renal epithelial to mesenchymal epithelial transition (EMT). HPE was used in the glucose-incubated HK-2 cell line at 0.1, 0.5, and 1.0 mg/mL concentrations. It inhibited, in the dose-dependent manner, the expression of angiotensin II receptors (AT-1) and, thus, regulated by it transforming growth factor β1 (TGF-β1), and vimentin, which is downstream to TGF-β1. This indicates that HPE prevents EMT. What is more, the authors revealed that HPE restored the elevated expression of a fibroblast differentiation marker—fibronectin, and recovered the expression of E-cadherin improving the cell junctions among renal tubular cells [93]. In another study, a different mechanism connected to EMT and diabetic nephropathy, namely insulin resistance, was examined. The authors who tested 1.0 mg/mL concentration of HPE in the HK-2 cells incubated in high-glucose observed that HPE inhibited the expression of type 4 dipeptidyl peptidase (DPP-4), which was elevated by high-glucose environment. That, in turn, resulted in a decrease of insulin receptor substrate-1 (IRS-1 (S307)) phosphorylation level, and thus vimentin expression. The expression level of phosphatidylinositol 3-kinase (PI3K), which was significantly decreased in high-glucose condition, also was restored after HPE treatment. The phosphorylation of IRS-1 (S307) is a typical sign of insulin resistance, while PI3K is connected to insulin sensitivity, thus, HPE may counteract diabetic nephropathy by alleviating insulin resistance and subsequent EMT [94]. Another study, also conducted on the HK-2 cells, but treated with palmitate instead of glucose, also focused on HPE effect on nephropathy resulting from insulin action alterations. Similar to the HK-2 cells that were incubated in high-glucose medium [94], HPE beneficially affected the palmitate-stimulated HK-2 cells. After treatment with HPE at a concentration of 1.0 mg/mL, the level of phosphorylated IRS-1 and, consequently the expression of AT-1 and vimentin decreased in these cells, while phosphorylation of PI3K increased. Also, HPE enhanced the insulin-stimulated glucose uptake in these cells. All these results suggest that HPE protects against nephropathy via insulin sensitivity regulation (Figure 1) [95].

Roselle was also demonstrated in vitro as a protective agent against high-glucose-induced vascular lesions. The study was conducted on the vascular smooth muscle cells (VSMC), more precisely on rat aortic smooth muscle A7r5 cells, which were incubated in a high-glucose medium with an addition of *Hibiscus sabdariffa* polyphenolic isolate (HPI) at different concentrations (0.01 mg, 0.1 mg, and 1.0 mg HPI) [30]. The study revealed that HPI reduced the growth and migration of VSMC, and this effect was dose- and time-dependent. It was shown that HPI suppressed the elevated expression of connective tissue growth factor (CTGF) and downstream processes regulated by it, including the activation of an enzyme involved in cell migration—matrix metalloproteinase 2 (MMP-2) as well as a receptor of advanced glycation end product (RAGE) expression and RAGE-mediated expression of proliferating cell nuclear antigen (PCNA), which is a marker for of cell proliferation. Moreover, apart from suppressing the whole CTGF signaling cascade, HPI also revealed the potential to regulate the expression of each of these genes connected to atherosclerosis and VSMC migration and proliferation as well as to glycation separately. These results indicate that roselle polyphenolic isolate may be a promising agent for protecting blood vessels in diabetic conditions (Figure 1) [30].

Diabetic foot is a common complication of diabetes. Its formation may be caused by neuropathies, circulatory disorders, as well as low resistance to microbes in diabetic patients. This condition can lead to many complications and in the worst case, amputation of the infected limb [96]. There is an in vitro study evaluating the antibacterial activity of roselle extracts against the most common diabetic foot pathogens [97]. Ethanolic extracts from roselle calyces and epicalyces at concentrations of 100 mg/mL and 200 mg/mL were analyzed against clinically-isolated bacteria strains. Based on minimal inhibitory concentration (MIC) results it was concluded that both doses have antimicrobial activity towards *Klebsiella pneumoniae*, *Staphylococcus aureus*, *Staphylococcus epidermidis*, *Escherichia coli*, *Proteus mirabilis*, and *Pseudomonas aeruginosa*. The highest MIC result for the extract with a concentration of 200 mg/mL was present for *Staphylococcus epidermidis* (9.5 ± 0.71 mm) and the lowest for *Klebsiella pneumoniae* (6.25 ± 0.35 mm). For the 100 mg/mL solution, the highest MIC was 7.5 ± 0.71 mm for *Staphylococcus aureus*, and the lowest was 5.75 ± 0.35 mm for *Klebsiella pneumoniae*. What is more, roselle extracts exerted biofilm eradicating properties for all the tested bacteria. Also, the antibacterial effect of roselle was enhanced by combining the extract with apple vinegar. Thus, roselle was proposed as an effective and cheap natural medical agent to be used in diabetic foot infections (Figure 2) [97].

As roselle is not just calyces, other parts of this plant are also proven to reveal therapeutic effects (Figure 2). There is research showing that methanolic extracts from roselle fruits are also able to inhibit α-amylase (IC_50_ = 79 µg/mL). Moreover, it is believed that they may have a beneficial effect on the wound healing process. These abilities are attributed to fibroblast stimulation as well as regulation and coordination of wound vascularization by methanolic extracts from roselle fruit [98]. Also, methanolic extracts and fractions of various solvents (butanol, ethyl acetate, and n-hexane) prepared with roselle fruit were tested with regard to insulin secretion stimulation and glucose uptake [62,99]. The studies performed on rat pancreatic β-cell BRIN-BD11 line showed an indirect hypoglycemic effect of fruit methanolic extracts and fractions by stimulating insulin secretion in these cells, but n-hexane and butanol fractions exhibited a higher cytotoxic effect on the tested cells than methanolic extract or ethyl acetate fraction [62]. Glucose uptake by 3T3F442A adipocytes and regulation of glucose transporter 4 (GLUT4) in L6 myotube cells was also assessed for the aforementioned fruit extracts [99]. The glucose uptake by 3T3F442A adipocytes was stimulated by methanolic extract and fractions prepared from *Hibiscus sabdariffa* fruits, but these preparations did not have a significant impact on GLUT4 distribution on L6 myotubes surface [99]. The authors conclude that the fruits of roselle provide a promising basis for the synthesis of a new generation of antidiabetic drugs (Figure 2) [62]. Sunmonu et al. [100] confirmed the anti-diabetic properties of another part of the roselle plant. The authors investigated aqueous, ethanolic, and methanolic extracts from the roselle stem bark. Each of the extracts showed the ability to inhibit the activity of α-amylase, and the aqueous extract was the most potent, comparable with acarbose 46.79 ± 1.80 (percentage α-amylase inhibition relative to acarbose), while the methanolic extract revealed lower potential to inhibit this enzyme 17.11 ± 1.01 (percentage α-amylase inhibition relative to acarbose) [100].

All the above-mentioned studies indicate that roselle extracts are effective at the in vitro stage, and thus can be promising agents in the treatment and prevention of diabetes and its complications also in the in vivo set-ups.

## 4. *Hibiscus sabdariffa* in Diabetic and Pre-Diabetic Animal Studies

### 4.1. Hibiscus sabdariffa and Its Hypoglycemic Effects in Animal Studies

Roselle and its multidirectional pharmacological properties in diabetes are the subject of many studies conducted not only at the in vitro level but also in animal models [19]. There are many reported experiments performed in mice and rats showing that different types of roselle extracts reveal an antihyperglycemic effect (Figure 2) [7]. Aqueous extracts obtained from flowers or calyces of roselle were described as hypoglycemic agents. This was proven in research conducted by Ibrahim et al. [61]. The authors administered aqueous extract that was prepared with dried roselle calyces at a dose of 500 mg/kg b.w. to the rats in which diabetes was induced with 60 mg/kg of streptozotocin. Roselle was administered once daily via oral gavage for four weeks post-induction of diabetes. The results showed that the administration of this extract was comparable to the effect of glibenclamide administered at a dose of 600 mg/kg and significantly lowered the level of fasting blood glucose compared to diabetic, untreated rats. Nevertheless, the glucose level was still higher in both these intervention groups than in the non-diabetic rats. In this study, roselle extract was also combined with glibenclamide, and this treatment resulted in a significant fasting blood glucose level reduction to the level that was observed in non-diabetic animals [61]. A similar observation regarding the hypoglycemic effect of roselle was made in other studies, where diabetes was induced in rats by 45 mg/kg of STZ. These rats were treated with an aqueous extract from roselle calyces at a dose of 100 mg/kg b.w. administered orally for 28 days, and this treatment significantly reduced blood glucose levels [101,102]. The authors suggest that this effect may be through the stimulation of β-cells of islets of Langerhans secretion of insulin or enhanced transport of blood glucose to the peripheral tissues [101]. The hypoglycemic effect of aqueous extracts from roselle calyces was also confirmed in a study performed on rats with diabetes that was induced by a single administration of alloxan at a dose of 150 mg/kg b.w. [103]. These diabetic rats were orally administered with roselle calyx extract at a dose of 30 mg/mL ad libitum for 21 days, and it turned out that the treatment resulted in significantly lower blood glucose levels in comparison to the untreated rats [103]. Similar conclusions were reached in research by El-Hady et al. [104]. In a rat model of alloxan-induced diabetes mellitus, they tested the effect of cold and hot roselle calyces extracts. The animals were orally treated with 250 mg/kg b.w. of both types of extracts for four weeks. The results indicate that both extracts significantly lowered blood glucose levels in these rats [104]. In another study, the effect of roselle steeping on glycemia was analyzed. The roselle calyces were steeped in 80 °C hot water for 3 min, and such preparation was administered to alloxan-induced diabetic rats at a dose of 2 mL/200 g b.w. by an oral rat sonde instrument for 14 days. This study showed that roselle steeping significantly lowered the fasting glucose level and glucose level 2 h after the meal compared to the untreated rats and to glucose levels recorded before the roselle treatment period [66]. It was also presented in another research that aqueous extracts from roselle calyces improve glycemia in rats with diabetes induced by STZ at a dose of 65 mg/kg based on an oral glucose tolerance test (OGTT). Administration of these extracts at the doses of 200 and 500 mg/kg b.w., using oral intubation, resulted in lowering blood glucose levels at subsequent measurement time points. The glucose level in the case of the extract with a higher concentration was lower at each measuring point compared to the extract with a lower concentration. This test was performed on the last day of the eight-week-lasting experiment [105]. Interestingly, Giacoman-Martínez et al. [24] showed that aqueous extracts from the roselle calyx did not reduce the level of glucose in the OGTT in any way. However, a dichloromethane extract from roselle calyces demonstrated the ability to lower glucose level in this test. The experiment was performed on healthy mice. Fifteen minutes before glucose administration, the appropriate groups were administered with 200 mg/kg b.w. aqueous extract and 200 mg/kg b.w. extract of dichloromethane, orally. After this time, the animals were administered glucose at a dose of 2 g/kg b.w. [24]. There are also studies in which aqueous extracts or infusions made with roselle affected the insulin level [34,67,101,102,104,106]. Some authors suggest that gallic acid from roselle is responsible for increased insulin secretion through the regeneration of β islets in the pancreas, which in turn improves insulin sensitivity and reduces insulin resistance [31,94,95]. Interestingly, aqueous extracts of roselle were able to indirectly alleviate insulin resistance by reducing body fat. In a study performed on obese rats, roselle aqueous extracts administered at doses of 250 and 500 g/kg b.w. were shown to reduce the AUC in the OGTT and Homeostatic Model Assessment—Insulin Resistance Index (HOMA-IR) in the tested animals, reduced glucose absorption in duodenum, and increased glucose uptake in gastrocnemius muscle and abdominal adipose tissue. It also inhibited lipid accumulation by increasing lipoprotein lipase activity and reducing the expression of adipogenesis genes. This led to a reduction in adipose tissue mass in these rats and a reduction in the level of pro-inflammatory cytokines and the oxidative state. As a result of these processes, insulin resistance was reduced, and tissue sensitivity to insulin increased [89]. In a rat model of insulin resistance induced by a high-fructose diet, an increase in insulin sensitivity was also demonstrated. The animals were orally administered 500 mg/kg b.w. of roselle aqueous extracts for four weeks. The results obtained at the end of the study show that these extracts improved insulin sensitivity, as depicted by reducing the HOMA-IR, and reducing the AUC in the OGTT test compared to the untreated rats [107]. There are also reports describing the beneficial effects of roselle calyces aqueous-ethanolic solutions on HOMA-IR in rats fed with a high fructose diet. The solutions were orally administered at the doses of 100, 200, and 400 mg/kg b.w. for five weeks to the rats, and the two higher doses decreased HOMA-IR values, while the most significant decrease occurred at the highest dose [106]. The effectiveness of lowering the glucose level and increasing the insulin level in the blood after administration of aqueous extracts from roselle calyces in the metabolic syndrome is also confirmed by other sources. There is a study in which rats with fructose-induced metabolic syndrome were treated for eight weeks with roselle calyces aqueous extract at the doses of 100 and 200 mg/kg b.w. administered orally. The employed treatment significantly decreased the blood glucose level, serum insulin level, and the HOMA-IR index [108]. The beneficial effect of aqueous infusions from roselle calyces on HOMA-IR was also presented elsewhere. Administration of 100 µL of such infusion significantly reduced the HOMA-IR index in rats with artificially induced metabolic syndrome [67].

Numerous studies concern ethanolic extracts obtained from roselle flowers and calyces. Ethanolic extract prepared from roselle flowers was shown to reduce blood glucose levels in rats with diabetes induced by intraperitoneal injection with alloxan at a dose of 100 mg/kg. The animals were treated daily with the extracts at doses of 100 and 200 mg/kg b.w. for four weeks. Both doses significantly reduced the blood glucose levels as compared to the control group, but it was observed that the higher concentration of extract showed a greater ability to lower glucose levels [109]. In another study performed in rats, but with diabetes induced with streptozotocin at a dose of 55 mg/kg, the 8-week-lasting via oral force-feeding administration of 100 mg/kg of ethanolic extract prepared from the roselle calyces was also demonstrated to reduce the plasma glucose level in comparison to the diabetic, untreated rats [110]. A rat diabetic model was also used in order to compare the antidiabetic effect of ethanolic and aqueous extracts prepared with roselle calyces. Diabetes in these rats was induced with 45 mg/kg of streptozotocin, and the animals were treated orally with both types of extracts at doses of 200 and 400 mg/kg b.w. for eight weeks. The obtained results suggested that both extracts at both concentrations reduced the serum glucose level in comparison to the control diabetic animals in the intraperitoneal glucose tolerance test (IPGTT). Also, the insulin tolerance test performed on day 54 of the experiment gave promising results. Both types of extracts have been shown to increase the sensitivity to exogenously administered insulin at both doses [111].

Ethanolic extracts from roselle flowers and calyces also are described to reduce blood glucose level in diabetic mouse models. One of the published studies revealed that the administration of ethanolic extracts prepared with roselle calyces at the doses of 200, 400, and 600 mg/kg b.w. by oral sonde, for five weeks to the mice with diabetes induced with streptozotocin at a dose of 55 mg/kg resulted in significantly lowered blood glucose level and this effect was dose-dependent. The authors of this research also tested other types of extracts—prepared with n-hexane and ethyl acetate, but they did not observe such a prominent effect on the blood glucose levels as noticed for ethanolic extracts [112]. Interestingly, the ethyl acetate fraction from ethanolic roselle extracts also revealed a hypoglycemic effect [113]. Seung et al. [113] used a mouse diabetes model, in which diabetes was induced with a single intraperitoneal administration of STZ at a dose of 150 mg/kg b.w. The results of their work showed that in the intraperitoneal glucose tolerance test, the roselle extracts administered orally by gavage at a doses of 100 and 200 mg/kg b.w. lowered the blood glucose level in relation to the diabetic, untreated mice. This test was performed after four weeks of feeding the animals with the extracts and the results may suggest that roselle and its extracts are effective in lowering fasting and postprandial glucose levels [113].

Other types of roselle extracts which were extensively examined are flavonoid-rich or polyphenolic extracts (HPE) from calyces or flowers. The first type of extract is obtained by extracting plant material with 70% methanol, then methanol is evaporated; then the aqueous phase is partitioned by ethyl acetate, followed by freeze-drying of the aqueous fraction which is used for further studies [114]. On the other hand, HPE are extracts prepared by a specific protocol described in different publications. In brief, firstly the plant material is extracted with methanol; then the eluent is evaporated, suspended in water, and then partitioned successively with n-hexane and ethyl acetate; then once again evaporated to dryness [65,93,110,115,116,117].

The beneficial effect of flavonoid-rich extracts from roselle calyces was demonstrated in the T1DM diabetic model of rats, in which diabetes was induced by 80 mg/kg streptozotocin injection. In this study, the extract was administered in daily doses of 1.75 g/kg of extract dissolved in 1.7 mL/kg of distilled water and administrated orally by gavage for 15 days. Such treatment resulted in hypoglycemic and serum insulin-elevating effects. The extracts significantly reduced the blood glucose levels and significantly increased the serum insulin levels compared to the diabetic untreated animals [114]. The hypoglycemic effect of polyphenolic extracts from roselle calyces was demonstrated by Peng et al. [117]. These authors proved that, in rats with T2DM induced by a single injection of STZ at a dose of 35 mg/kg b.w. and a high-fat diet, polyphenolic extracts of roselle calyces orally administered at the doses of 100 mg/kg and 200 mg/kg were able to lower serum glucose levels as compared to the glucose levels reported in the serum of the untreated animals. Moreover, they showed in their research that these extracts were able to alleviate hyperinsulinemia, which is a common symptom of T2DM. This may prove that roselle extracts have a potential effect against insulin resistance [117]. Other sources also indicate the effectiveness of polyphenolic extracts from roselle calyces in lowering blood glucose levels in rats in which diabetes was induced by STZ at a dose of 55 and 60 mg/kg b.w. In all these studies, this extract was administered daily at doses of 100 mg/kg or 150 mg/kg b.w. orally (by gavage or force-feeding) for four weeks [65,115,118,119,120,121].

The literature also describes the therapeutic use of roselle in diabetes mellitus in combination with other products. In studies on diabetic rats, it was shown that the combination of aqueous extracts from 300 g of fresh leaves, stem, and roots of *Hibiscus sabdariffa* + 100 g of *Allium sativum* + 100 g of *Zingiber officinale* administered orally daily for 17 days had a greater effect in lowering the blood glucose levels than the use of roselle aqueous extracts alone [122]. The synergistic effect of aqueous extracts of roselle combined with *Zingiber officinale* has also been described by Agoreyo et al. [123]. Both extracts have been shown to have a significant hypoglycemic effect and are even stronger when they are administered together. However, the animal model was the main difference from the above-described study performed by Ojewumi et al. [122]. The animals were not diabetic but were fed with breeding pulp or a combination of breeding pulp (99%) and cholesterol (1%) [123]. On the other hand, the results from a study investigating the combined effects of hydroalcoholic extracts from roselle flowers and *Carum carvi* were not so promising. Separately, both extracts were capable of lowering the blood glucose level of diabetic rats, but their joint action was not synergistic. Roselle extracts show a greater ability to lower glucose levels than *C. carvi* extracts [59]. Ethanolic macerates of roselle flower petals were also tested in combination with *Physalis angulata* in alloxan-induced diabetic rats. The results of the experiment showed that the administration of roselle and *Physalis angulata* combination at the doses of 10 mg/200 g b.w. and 30 mg/200 g b.w, respectively, lowered the blood sugar level of the tested animals with a power similar to that of glibenclamide at a dose of 0.052 mg/200 g b.w. In addition, the extract combination improved the activity of Langerhans islets, which was shown in the histopathological examination [124]. An interesting fact is that aqueous extracts of roselle, used as an additive in fermented milk, enhance its hypoglycemic and hypolipidemic effects [125]. Similar observations were made in a study assessing the addition of an aqueous roselle calyx extract to the probiotic goat milk yogurt. Such a combination increased the hypoglycemic activity in an in vitro assay of α-glucosidase inhibition, but this inhibitory activity of the roselle-enriched probiotic yogurt decreased over storage time (Figure 2) [126].

It is also worth mentioning that the increasing hyperglycemia and insulin resistance accompanying diabetes may contribute to the development of oxidative stress [13]. Too high blood glucose level and oxidative phosphorylation increase ROS production in the mitochondria [13,127]. This, in turn, creates or increases the already existing oxidative stress in the endoplasmic reticulum in the β-cells of the pancreatic islets. In addition, oxidative stress in the endoplasmic reticulum itself causes ROS formation and may contribute to ROS formation in the mitochondria. Briefly, ROS in the mitochondria induces oxidative stress in the endoplasmic reticulum, and oxidative stress in the endoplasmic reticulum induces ROS formation in the mitochondria, thus creating a vicious circle. As a result of this process, the β-cells of the pancreatic islets cannot respond appropriately to the increased blood glucose levels. Moreover, ROS damages other cellular components, lipids, proteins, and DNA [127]. Interestingly, lipid peroxidation products, especially malondialdehyde, show cytotoxic, mutagenic, and carcinogenic properties [128]. The studies indicate that the administration of roselle petal extracts to rats with T2DM at doses of 195 mg/200 g b.w. and 260 mg/200 g b.w. for 21 days can effectively reduce the level of malondialdehyde and thus reduce its adverse effects [129]. The ability to significantly reduce malondialdehyde levels by aqueous roselle extracts was also confirmed in other studies. However, it should be noted that this effect was greater when the roselle extract was combined with glibenclamide [61]. Damage to cell components can lead to transcriptional changes such as insulin resistance [127,130]. The resulting insulin resistance leads to hyperglycemia, which will also contribute to the formation of an increased amount of ROS [127]. Thus, oxidative stress has a two-way mode of action in diabetes. It weakens insulin secretion and reduces the response of the body’s tissues to its actions [131], consequently leading to the formation of T2DM [130]. Interestingly, it has been noticed that the metabolic pathways that contribute to the increased formation of oxidative stress in diabetics are the pathways of sugar and fat metabolism. These include glycolysis, the advanced glycation end-product pathway, the hexosamine pathway, and the polyol pathway [131]. There are in vitro and in vivo studies confirming the beneficial effects of aqueous and ethanolic extracts from roselle calyces. The antioxidant activity of these extracts was demonstrated in a 5-week study in rats with alloxan-induced diabetes [132]. The effect of roselle extracts on oxidative stress and ROS, as well as their potential therapeutic effect in selected organs, is described in the following chapters of this review.

### 4.2. Hibiscus sabdariffa and Pancreatic Support

In diabetes, especially T1DM, there is an autoimmune destruction of the β-cells of the Langerhans islets in the pancreas. Consequently, it reduces or even inhibits insulin secretion by these cells [133]. In T2DM, the failure of β-cells’ function is also observed, not only because of the death of these cells but also due to the changes in their phenotype and mass [134,135]. However, based on many studies conducted on T2DM patients and animals, it can be concluded that the β-cell dysfunction may be reversible [134]. In a study performed on rats with STZ-induced T2DM treated with an ethanolic extract from roselle calyces, a beneficial effect on the pancreas was revealed. The rats were orally treated with the extract at doses of 0.1 and 1 g/kg b.w. According to histological examinations, it was demonstrated that the higher dose was able to increase the number of Langerhans islets in the pancreas. Moreover, the higher dose of roselle calyces ethanolic extract increased the concentration of insulin in the serum of the rats and improved glucose tolerance measured in the OGTT [136]. Interestingly, the literature also mentions that flavonoid-rich aqueous fraction of methanolic extract of roselle calyces could exert a similar effect in T1DM rats in which diabetes was induced with 80 mg/kg of STZ. The study revealed that this extract orally administered by gavage at a dose of 1.75 g/kg b.w. for 15 days improved the volume of pancreatic islets and increased the number of β-cells. The ability to protect and regenerate pancreatic islets is probably attributed to the antioxidant properties of roselle [114]. Roselle methanolic extract was also able to act on the glucagon-like peptide-1 (GLP-1) hormone [137]. This hormone, secreted in the ileum, plays an important role in the pancreas by increasing insulin secretion, increasing proliferation, and preventing pancreatic β-cell apoptosis [138]. There is a report in which STZ-induced diabetic rats (35 mg/kg of STZ was used) were treated with roselle methanolic extract at the doses of 200 and 500 mg/kg orally once a day for five weeks. In this study, the extract, especially the higher dose, improved the secretion of GLP-1 and maintained its concentration in the ileum at a level similar to that in healthy animals. Regarding pancreatic levels of GLP-1 in these rats, both the doses increased the level of this hormone compared to the diabetic control rats. This is presumed to be due to roselle active compounds, such as leucoside, which binds to sodium-glucose co-transporter-1 (SGLT-1). This in turn leads to increased GLP-1 secretion. Moreover, delphinidin-3-sambubioside from roselle may also act as a GLP-1 analog by interacting with the pancreatic GLP-1 receptor (Figure 1) [137].

### 4.3. Hibiscus sabdariffa and Anti-Lipidemic Activity

Apart from elevated blood glucose levels, diabetes is also accompanied by other dysfunctions. One of them may be disorders of fat metabolism—dyslipidemias, and more precisely, an increase in triglycerides, cholesterol, and low-density lipoprotein (LDL) and also a decrease in the amount of high-density lipoprotein (HDL) lipoproteins [139,140]. The reason for the occurrence of these pathologies is the phenomenon of oxidative stress caused by the presence of reactive oxygen species (ROS) in the tissues [141]. Therefore, in diabetes mellitus, it appears to be beneficial to prevent the occurrence of excessive amounts of ROS. Due to their hypoglycemic, antioxidant, and antihyperlipidemic effects described in the literature, roselle and its products may be a solution to this problem [117]. Farombi et al. [109] investigated the anti-lipidemic and antioxidant properties of ethanolic roselle flower extracts. They conducted the experiment in a rat model of alloxan-induced diabetes. The researchers demonstrated the effect of the extracts on plasma lipid profiles. Both lower (100 mg/kg) and higher (200 mg/kg) concentrations of extracts were able to lower the concentration of cholesterol, low-density lipoprotein (LDL), and very-low-density lipoprotein (VLDL) in the plasma. Moreover, both extracts showed the ability to increase HDL concentration, but not significantly. In addition, they showed in their research that ethanolic extracts from roselle flowers could moderate the decrease in GSH levels induced by alloxan, which confirms the antioxidant effect of roselle, and indirectly it improves lipid metabolism. Researchers also suspect that polyphenolic compounds such as dihydrobenzoic acids, including protocatechuic acid, may be responsible for the described activity of the plant [109]. Peng et al. [117] reached a similar conclusion in their research in which they investigated the effect of polyphenolic extracts from roselle calyces in streptozotocin-induced T2DM rats. In addition to the hypoglycemic effect described above, these extracts also reduced the triacylglycerol level in the serum, cholesterol, and the LDL/HDL ratio. Moreover, the tested extract significantly reduced the increase of lipid peroxidation, which illustrated its antioxidant effect [117]. Polyphenolic roselle calyx extract orally administered at the doses of 100 and 200 mg/kg b.w. was shown to normalize the lipid profile measured in the plasma of diabetic rats and the higher dose seemed to be more effective [116]. Aqueous extracts from roselle calyces also showed anti-lipidemic properties. It was confirmed in rats with alloxan-induced diabetes mellitus. The oral administration of extracts at a dose of 2 mL/200 g b.w. for 14 days significantly lowered triglyceride levels and increased blood HDL levels compared to the results before the study [66]. Interestingly, it was specified which compound of roselle may have an effect in preventing obesity—one of the factors predisposing to T2DM development. It is delphinidin-3-sambubioside, which was administered by gavage to the HFD-induced obese rats at the doses of 15 and 30 mg/kg for eight weeks. In vivo studies for this anthocyanin were in line with the results obtained during the in vitro stage—it alleviated hyperlipidemia by affecting the regulation of target genes related to β-oxidation and lipogenesis of fatty acids through the regulation of 5 ‘AMP-activated protein kinase in the liver of obese rats: PPARα, CPT1 were upregulated, SERBP-1c and its downstream FASN as well as acetyl-CoA carboxylase (ACC) were downregulated. Moreover, the expression of acyl-coenzyme A oxidase (ACOX) involved in fatty acid β-oxidation was restored. What is more, the expression of mRNA for two enzymes taking part in cholesterol catabolism (CYP7A1) and synthesis HMGCR were also assessed. The expression of CYP7A1 was higher after the treatment, while HMGCR was lower (Figure 1). This evidence points to the potential use of roselle-derived phytochemicals in the treatment of obesity and hence the prevention of diabetes (Figure 2) [91]. The anti-obesity effects of roselle aqueous extract was also proven in a study on HFD-induced obese rats. These animals were administered orally with roselle extract at the doses of 250 mg/kg and 500 mg/kg b.w. for eight weeks, and this treatment reduced perirenal fat, epididymal fat and abdominal fat content, as well as the sum of mesenteric, perirenal, and epididymal content when compared to non-treated rats. Also, the duodenal glucose absorption was reduced, while glucose uptake in the muscle and abdominal adipocyte tissue increased after roselle treatment [89]. The beneficial effect of roselle extracts in diabetic animals on the lipid profile was also presented in other works [104,110,118,120,121,142,143].

### 4.4. Hibiscus sabdariffa and Hepatoprotective Effect

The liver and its function play a key role in regulating carbohydrate metabolism. The correct glucose level in the blood and organs is maintained thanks to its action [144,145]. Increased glycogen and lipid levels accompanying diabetes may contribute to fibrosis and cirrhosis of the liver, abnormal activity of liver enzymes, or impairments of the bile duct functioning in the liver [63,146,147]. The literature shows that roselle calyces ameliorate biochemical and histological changes in the liver of rats with diabetes STZ. The results showed that these extracts restored the levels and activities of glutathione, catalase, superoxide dismutase, glutathione peroxidase, aspartate aminotransferase, alanine aminotransferase, and alkaline phosphatase to the state before diabetes. In addition, histological examinations revealed a reduction in liver fibrosis and excess glycogen deposition. According to the authors, these effects were attributed to the presence of flavonoids and the antioxidant properties of these roselle extracts [148]. The aqueous extract from fresh roselle calyces administered orally also revealed a beneficial effect on the liver, as evidenced by the study presented by Husin et al. [101]. They have proven that aqueous roselle extracts are able to reduce the level of aspartate aminotransferase and alanine aminotransferase both in the liver and plasma of rats with diabetes. Additionally, it was shown that the extracts were able to significantly reduce the destruction of hepatocytes caused by diabetes [101]. Another beneficial effect of roselle extract on the liver was presented in a study in which *Hibiscus sabdariffa* ethanolic extract was administered to alloxan-induced diabetic rats. The extract was administered for four weeks at two doses: 100 and 200 mg/kg b.w. and lovastatin was used as a positive control. The study showed that the higher dose of the extract, similar to lovastatin, increased the activity of the phosphatidate phosphohydrolase—an enzyme involved in lipid metabolism—in the liver, with a simultaneous decrease in the levels of hepatic lipids and lipid oxidation marker, malondialdehyde. Also, both the doses exhibited an antioxidative effect in the liver [109]. A study was also performed on the HFD-induced obese hamsters fed with a HFD diet containing roselle aqueous extract (HSE) and a polyphenolic fraction (the HPE diets contained 0.1 or 0.2% of HSE or HPE). Both HSE and HPE reduced the cholesterol and triglyceride content in the liver of the tested animals, but HPE seemed to have a better effect [90].

### 4.5. Hibiscus sabdariffa and Kidney Protective Effect

Diabetic nephropathy is another comorbid condition of diabetes mellitus. There are multiple molecular pathways that are included in the development of this diabetes-related kidney disease [149]. The effect of roselle aqueous extract on nephropathy in rats with STZ-induced diabetes was also investigated. Additionally, the obtained results were compared with that of angiotensin-converting enzyme inhibitor drug—lisinopril, and the combined effect of lisinopril with roselle was also tested. The results of the study showed that daily 8-week oral administration of roselle extracts at the doses of 100, 200, and 400 mg/kg b.w. improved diabetic nephropathy. It reduced inflammation by suppressing the toll-like receptor 4/nuclear factor kappa-light-chain-enhancer of the activated B-cells/TNF-α (TLR 4/NF-κB/TNF-α) pathway, thus, reducing the inducible NO synthase (iNOS) expression, lowered oxidative stress, as well as decreased the TGF-β levels (Figure 1) induced by STZ. Interestingly, the extract at a concentration of 400 mg/kg b.w. showed an effect comparable to lisinopril at a dose of 5 mg/kg b.w. However, their correlated effect at doses of 2.5 mg/kg b.w. lisinopril and 100 mg/kg b.w. roselle extract significantly improved the symptoms of nephropathy; it was more potent than the action of lisinopril or roselle extract alone. Therefore, the results suggest using an aqueous extract from roselle calyces as an independent-action equivalent of angiotensin-converting enzyme inhibitors or as an adjuvant of conventional drugs [150]. Wang et al. [142] demonstrated in their studies conducted on a rat model of STZ diabetes that tube-feeding with aqueous extracts of dried roselle flowers at the doses of 100 and 400 mg/kg b.w. for eight weeks can alleviate diabetic nephropathy. The proposed mechanism of roselle action is by improving the oxidative state in the kidneys and thus regulating the protein kinase B/BCL2-associated agonist of cell death/14-3-3γ (Akt/Bad/14-3-3γ) signaling. The authors proposed that roselle, by reducing the hyperglycemia-induced oxidative stress and high ROS levels while simultaneously enhancing the antioxidant enzymes activity, regulates this pathway by increasing the expression of phosphorylated Akt, phosphorylated Bad, and 14-3-3γ protein, and thus, NF-κB expression, mitigating the renal tubules apoptosis (Figure 1) [142]. They also confirmed that by significantly increasing the activity of catalase and glutathione level in the kidneys of rats, roselle is able to reduce lipid peroxidation in this organ [142]. There are also studies concerning the effect of polyphenolic roselle extract (HPE) on the kidneys of diabetic rats [93,94,95,116,151]. HPE orally administered at the doses of 100 and 200 mg/kg b.w. was able to prevent the occurrence of diabetic nephropathy in a rat model of type 1 diabetes. Normalization of BUN levels in the plasma, improvement the histological image of the kidneys (less hydropic changes were observed), and a reduction of the kidney weight was observed. What is more, this treatment increased the catalase activity and glutathione level with a simultaneous decrease of the malondialdehyde content in the kidneys of diabetic rats. Nevertheless, it should be highlighted that in the majority of the tested parameters, the higher dose was more effective [116]. Yang et al. in their studies [93,151], also observed a beneficial effect of HPE in diabetic nephropathy in STZ and HFD-induced type 2 diabetic rats. The authors observed that HPE administered to T2DM animals might prevent basement membrane disruption by restoring the altered type IV collagen expression in the kidneys [93]. HPE that was orally administered to type 2 diabetic rats at the doses of 100 and 200 mg/kg b.w. reduced albuminuria and clearance of creatinine. The higher dose of HPE reversed the nephritic changes observed in histological stainings, such as glomerular hypertrophy or fat deposition. Moreover, advanced glycation end-products (AGEs), glomerular cluster of differentiation 31 (CD31), and tubular connective tissue growth factor (CTGFs) expression in the kidneys was suppressed after treatment with HPE (Figure 1). Also, the level of lipid peroxidation in the kidney of the tested animals was reduced upon HPE administration. Histological analyses revealed that HPE prevented renal fibrosis by reducing collagen accumulation in the kidney of the diabetic animals [151]. Moreover, polyphenolic roselle extracts showed the ability to inhibit renal fibrosis by inhibiting the transition of the renal epithelium to the mesenchymal epithelium in the rat T2DM model [94,95]. These properties are based on the ability of HPE to inhibit dipeptidyl peptidase-4, which is involved in the transition of the renal to mesenchymal epithelium. Additionally, HPE reduced the markers of insulin resistance—insulin receptor substrate-1 and vimentin in the renal tubular region. Therefore, these extracts can be an adjuvant in preventing the occurrence of diabetic nephropathy [94,95]. Roselle may also be a promising agent in the treatment of renal dysfunction in metabolic syndrome. A 2% strong roselle infusion was administered in drinking water to the rats with experimentally-induced metabolic syndrome. It was observed that after treatment, the overall condition of the kidneys improved. This was illustrated by increasing the glomerular filtration rate, increasing creatinine clearance, and thus lowering the blood pressure. Additionally, it was shown that there was an increase in enzymatic and non-enzymatic antioxidant systems, which leads to the reduction of oxidative stress and, consequently, the reduction of the occurrence of renal impairment [67].

Diabetic nephropathy is one of the risk factors of cardiovascular morbidity and mortality in diabetic patients [152]. Al-Qahtani et al. [111] showed in their studies on a rat model of diabetes that aqueous and ethanolic roselle flower extracts that were orally administered could counteract the pathological changes in the levels of serological markers of kidney function, which also may contribute to cardiovascular disease development. Both extracts have the effect of lowering the concentration of urea, uric acid, and creatinine in the serum and have a positive effect on the lipid profile of the body. Thus, they can protect against the occurrence of renal dysfunction and indirectly prevent the occurrence of cardiovascular diseases [111]. Similar conclusions were reached by the authors of another study in which the aqueous extracts of roselle calyx were tested in a rat model of diabetes. In diabetic animals that were orally treated with roselle aqueous extracts, a decrease in serum creatinine concentration, an increase in the glomerular filtration of the kidneys by dilation of blood vessels, and a decrease in the level of lipids were noted. The hypoglycemic effect of roselle extracts was also confirmed. These data suggest that aqueous extracts of roselle lower the risk of kidney dysfunction by improving their functioning and parameters and indirectly reduce the risk of atherosclerosis and cardiovascular diseases (Figure 2), which will be discussed in the next chapter [105].

### 4.6. Hibiscus sabdariffa and Cardiovascular Protective Effect

One of the most common long-term complications of diabetes mellitus is cardiovascular disease. It manifests as systolic and diastolic dysfunction of the left ventricle, which may lead to heart failure [153,154]. The causes of these disorders are, among other things, the overproduction of ROS and, consequently, oxidative stress; cardiomyocyte fibrosis or apoptosis; or mitochondrial dysfunction [153,154,155]. The literature shows that polyphenolic extracts from roselle calyces were able to prevent the changes involved in diabetes-related cardiovascular disorders (Figure 2). In a rat model of STZ diabetes, these extracts were shown to alleviate oxidative stress by enhancing the mitochondrial antioxidant protection system after oral administration. Moreover, they had a positive effect on dyslipidemia and reduced the state of hyperglycemia. Therefore, they may reduce the likelihood of heart and vascular diseases [121]. There are also reports indicating that roselle polyphenol-rich extract (HPE) contributed to the improvement of the heart condition in type 1 diabetic rats, in which diabetes was induced with streptozotocin at a dose of 55 mg/kg b.w. HPE oral supplementation to these animals led to a reduction in the level of oxidative stress by increasing the activity of antioxidant enzymes as well as reducing the level of cardiac markers of oxidative stress. What is more, treatment with HPE resulted in a reduction of cardiomyocyte hypertrophy and fibrosis and counteracting the diabetes-induced ultrastructural changes in the mitochondria. This extract also regulated dyslipidemia and improved the cardiac functions such as pressure in the left ventricle and cardiac contractility and relaxation rate, which in turn positively affected the coronary flow [110]. The effect of HPE on blood pressure was also demonstrated. According to the data obtained from the studies conducted on rats with diabetes induced with a high dose of STZ, in which systolic and diastolic blood pressure, as well as the mean arterial pressure, were measured, HPE turned out to be a hypotensive agent [65,120]. Some authors suggest that the antihypertensive effect of roselle is due to two of its two main anthocyanins—delphinidin-3-O-sambubioside and cyanidin-3-O-sambubioside. These two phytochemicals were shown to inhibit in vitro the activity of angiotensin-converting enzyme (ACE) by competing with its active site (Figure 1) [156]. The literature also points out that HPE is a vascular protective agent. It was demonstrated that HPE added to the diet at the doses of 100 and 200 mg/kg b.w. resulted in the suppression of RAGE and CTFG formation in the aortas of type 2 diabetic rats, thus can be a potential agent preventing diabetic vasculopathies (Figure 1) [117]. It also improved the structure of vascular smooth muscle cells altered by diabetes [65,118]. Moreover, thanks to its antioxidant properties, it was able to positively affect blood vessels, especially by the ability to prevent oxidative damage to the proteins and lipids within the aortas of the diabetic animals [65,118,120], increasing the depleted aortal GSH levels [118,120] and in some cases, increasing the activity of antioxidative enzymes in the thoracic aorta [118]. Moreover, in another study, the anti-inflammatory effect of aqueous extracts from roselle calyx on the aortas of the STZ-induced T2DM rats was examined. The extracts at the doses of 100, 200, and 400 mg/kg b.w. were administered by oral gavage for eight weeks. The study showed that the expression of inflammation markers: TLR 4, TNFα, and iNOS were enhanced in the aortas of the diabetic animals, while the expression of endothelial NO synthase (eNOS) was depleted. The administration of roselle extracts counteracted these alterations, which suggests that it protects blood vessels via an anti-inflammatory mechanism (Figure 1) [157].

Aqueous extracts from roselle calyces also revealed their beneficial properties on the circulatory system [157,158,159]. It has been proven in the T2DM rat model. The animals were orally administered 500 mg/kg b.w. aqueous extract of roselle, once daily for eight weeks. The study results show that the described treatment regimen caused a significant decrease in the diastolic ventricular pressure and an increase in (-) dP/dt (rate of rise of left ventricular pressure) compared to the untreated rats. It also improved the lipid profile and glycemia and alleviated oxidative stress in the cardiac tissue. Interestingly, treatment with the roselle extract did not affect the systolic and diastolic blood pressure [159]. Another study showed a protective effect of aqueous extracts on the oxidative stress of red blood cell membranes. Rats with diabetes induced by STZ served as the model. Roselle aqueous extract reduced the diabetes-induced oxidative damage to the red blood cell membrane lipids and proteins, as well as it increased the superoxide dismutase activity. Also, there were no abnormal or hemolyzed red blood cells visible in the smears that were prepared from the blood of diabetic animals. These results suggest that force-feeding with aqueous extracts from roselle calyx can also be effectively used as a supplement to the treatment of diseases associated with diabetes [158].

### 4.7. Hibiscus sabdariffa and Protective Effect on Fertility

Male diabetic patients suffer from hyperglycemia-related subfertility and/or infertility. The exact mechanisms underlying these impairments are not entirely understood. Some authors suggest that diabetes induces oxidative stress, resulting in oxidative damage, sperm DNA fragmentation, and the formation of glycation end products at the testicular level [160,161]. In experimentally-induced diabetic animals, infertility is connected with enhanced ROS production and lipid peroxidation in the testes, as well as with disturbance in endogenous antioxidative machinery, including SOD and CAT activities [162,163].

The literature shows that HPE from the roselle calyces effectively protected the testes of STZ diabetic rats (Figure 2). It was probably due to the ability of HPE to alleviate oxidative stress and its antihyperglycemic effect [119]. Similar conclusions were also reached by Kasim et al. [115]. The authors demonstrated that oral HPE supplementation for 28 consecutive days to diabetic rats improved testicular damage caused by oxidative stress, and counteracted oxidative damage to sperm. It was concluded that this effect was caused by the high content of polyphenols and other antioxidants in roselle. However, the exact mechanism of action is unknown [115]. There are also reports indicating that not only HPE improves the fertility of diabetic animals, but also an aqueous preparation prepared from fresh roselle flowers exerts a beneficial effect on this impairment. In a rat model with STZ-induced diabetes, the researchers demonstrated that the oral administration of roselle aqueous extract from fresh flowers for 28 days reduced sperm defects caused by diabetes. It improved the quality of sperm and its mobility and positively affected the level of follicle stimulating hormone (FSH), but the mechanism underlying these changes is yet to be discovered [102]. The effect of aqueous roselle extracts on the quality of semen and testes (reducing oxidative stress in them) has also been confirmed in another study, which was performed not in diabetic rats but in rats fed a high-fat diet [164].

T2DM, insulin resistance, and obesity may also be associated with disorders in the female reproductive system. They can manifest themselves, among others, in the form of polycystic ovary syndrome [165,166,167]. However, there is no literature data on the use of roselle and its products in such diseases.

### 4.8. Hibiscus sabdariffa and Its Effects on Other Organs

Diabetes mellitus is also associated with cognitive impairments. They arise as a result of, among other things, increased oxidative stress and the formation of advanced glycation end-products [168]. The beneficial effect of the roselle extract prepared by a specific extraction method (briefly, roselle ethanolic extract was evaporated in a vacuum, then consecutively separated with n-hexane, chloroform, and ethyl acetate, and the ethyl acetate faction was freeze-dried and used for the study) on neuronal disorders in diabetes was confirmed by Seung et al. [113]. The study was conducted in a diabetic mouse model induced by the administration of STZ at a dose of 150 mg/kg b.w. The extract was orally administered to the animals at the doses of 100 mg/kg b.w. and 200 mg/kg b.w. for four weeks. The treatment successfully relieved cognitive disorders, measured in multiple behavioral tests. The authors concluded that due to numerous polyphenolic components, roselle counteracts cognitive dysfunctions caused by diabetes through antioxidant activity and strengthening the cholinergic system (Figure 2) [113].

The anti-inflammatory effects of roselle extract were also evaluated in the spleen of the STZ-induced diabetic rats. Nevertheless, the extracts were administered orally for 21 days at the doses of 72 mg/200 g b.w. and 288 mg/200 mg b.w. but did not reveal an anti-inflammatory effect in the tested organ as the level of interleukin 6 (IL-6) did not change and the level of TNFα only tended to decrease [34].

In the course of diabetes, changes in the composition of bacterial species inhabiting the intestines are also observed in patients [169,170,171,172]. There are no reports in the literature regarding the influence of roselle extracts on the composition of the intestinal flora in the course of diabetes. However, as shown in studies by Diez-Echave P. et al. [173], phenolic extracts from roselle showed a prebiotic effect. The experiment was carried out in mice with obesity induced by a high-fat diet. The oral administration of roselle calyces extract at a dose of 25 mg/kg b.w. daily for 42 days reduced the ratio of *Firmicutes* to *Bacteroidetes* and modified the abundance of other genera and orders of microorganisms. This action resulted in the reduction of obesity-related intestinal dysbiosis; therefore, it can be concluded that these extracts show a prebiotic effect (Figure 2) [173]. However, in order to determine whether a similar effect of this raw material will also be observed in diabetic conditions, tests are necessary.

The eyes are also an organ that is negatively affected by diabetes. Therefore, there is a need to protect them during this disease [174]. No reports were found on the protective effect of roselle and its preparations on the eyes in the course of diabetic disease. However, there are studies that confirm the protective effect of anthocyanins from roselle extracts on the eyes of rats exposed to UVC radiation. The oral administration of anthocyanins at the doses of 2.5, 5, and 10 mg for 40 days to rats exposed to UVC for 4 h a day for 40 days counteracted the eye impairment that was caused by UVC radiation [175]. On the other hand, in a report conducted on subjects with normal eyes, it was revealed that consumption of a hot hibiscus drink negatively affected the tear film. The authors of this study hypothesized that high polyphenol levels in roselle might adversely impact the lipids and electrolyte content within the tear film [176]. Therefore, in order to determine whether roselle extracts reveal beneficial or deteriorating effects on the diabetic eye, experiments conducted in vitro or in vivo are necessary.

### 4.9. Hibiscus sabdariffa Is Not Only a Flower and Calyces

Apart from the calyces and flowers of roselle that are widely described in the literature, one can also find publications describing the beneficial effects of extracts from the leaves, stems, and seeds of this plant [54,60,177]. Studies show that methanolic roselle leaf extracts, when administered to rats with alloxan-induced diabetes, had an antidiabetic effect and lowered cholesterol and triglycerides levels compared to the untreated controls [60,177]. Similar properties were demonstrated for methanolic extracts from roselle stems [60]. Methanolic extracts from seeds also showed a hypoglycemic effect and reduced cholesterol, triglycerides, and LDL levels [54]. Therefore, it can be concluded that these plant parts are also effective in reducing the effects of hyperglycemia and dyslipidemia (Figure 2) [54,60,177].

### 4.10. Molecular Mechanisms Underlying Antidiabetic Effect of Hibiscus sabdariffa

The exact mechanisms of roselle’s beneficial effect are still unclear. Many scientists in their studies attempted to investigate the putative molecular pathways that are responsible for its action. Based on the literature data described above [24,30,31,87,89,90,91,93,94,95,109,117,142,150,151,156,157] we tried to graphically summarize the probable molecular pathways accountable for the positive effect of *Hibiscus sabdariffa* on diabetes and its complications. Since the calyces are the most commonly used part of this medicinal plant, only the mechanisms that were investigated in them are presented in Figure 1.

## 5. *Hibiscus sabdariffa* in Diabetic and Prediabetic Patients

The effectiveness of *Hibiscus sabdariffa* in the treatment of prediabetic and diabetic individuals was also documented in the trials involving humans (Figure 2) [53,55,109,178,179,180,181,182,183,184,185,186,187]. The hypoglycemic effect of roselle was confirmed in a quasi-experimental study performed by Mayasari et al. [187]. In this research, pre-diabetic women drank tea containing 5 g of roselle and 125 mg of stevia sweetener twice a day for 14 days. After two weeks of drinking such preparation, the fasting blood glucose level in the treated group decreased significantly compared to the untreated group. However, postprandial glucose levels measured 2 h after a meal did not change significantly in both the treated and untreated groups [187]. Similar observations regarding aqueous roselle extracts were made in the study in which elderly women with metabolic syndrome participated. In this research, the participants were treated with 150 mL of tea with 2 g of roselle twice a day for 21 days. The patients were monitored by health workers, and their standard medication regimen was not changed. This treatment resulted in a lower fasting blood glucose level, but the postprandial glucose level did not change when the results from the post-treatment measurements were compared with the pre-treatment values [182]. There was also a study in which a formulation containing *Hibiscus sabdariffa* and *Stevia rebaudiana* was prepared as a ready-to-drink product. It was evaluated in healthy patients in terms of safety for diabetic patients. It turned out that such a drink did not elevate the blood glucose levels, thus, the authors concluded that a drink made of roselle and stevia is safe for people with diabetes [188].

However, not every patient likes sour tea prepared from roselle. Therefore, capsules containing roselle extracts or roselle powder could be an alternative. Sarbini et al. [183] used capsules with 500 mg roselle powder in their studies on T2DM patients. Their research showed that consuming them twice a day for eight weeks significantly lowered the fasting blood glucose level compared to its level recorded before the treatment. The authors of this research also suggested that roselle may reduce the fasting insulin level and the HOMA-IR index, but there were no statistical significance in these parameters, and more studies are needed [183]. The hypoglycemic effect of roselle-containing capsules was confirmed in patients with metabolic syndrome. The patients in this study were divided into three intervention groups: treated with diet only, with capsules containing 100 mg of roselle ethanolic extract (HSEP), or with a combination of diet and the HSEP. The capsules were taken daily before breakfast for 1 month. The intervention with HSEP only lowered the blood glucose level in comparison to the level before the start of the treatment and improved several other parameters measured during the study, but the combination of diet and HSEP revealed better final results than the HSEP alone [179].

### 5.1. Effect of Hibiscus sabdariffa on Blood Pressure in Diabetic Patients

In addition to their hypoglycemic properties, roselle and its preparations also have a blood pressure-lowering effect. This was shown in studies by Abubakar et al. conducted on non-diabetic participants with 1 to 10% cardiovascular disease risk in 10 years [189]. A total of 25 men consumed 250 mL of roselle infusion with a high-fat meal. The research showed that roselle extracts could increase the concentration of nitrous oxide in the plasma, which may contribute to blood pressure reduction. However, more research is needed to confirm this thesis [189]. The antihypertensive activity of roselle was also confirmed in patients with T2DM with mild hypertension (inclusion criteria: minimum systolic blood pressure 160 mm Hg, minimum diastolic blood pressure 100 mm Hg). The patients drank 240 mL of water infusion from a 2 g sachet of roselle steeped for 20–30 min, two times a day for one month. After this time, the blood pressure measurement results showed a significant decrease in the systolic blood pressure in patients receiving roselle infusions compared to the untreated patients. Also, the mean pulse pressure decreased in the patients that were drinking the tea from roselle. However, diastolic blood pressure values did not change significantly compared to the untreated patients [190]. Mozaffari-Khosravi et al. reached similar conclusions in their research [178]. Patients with T2DM and mild hypertension consumed 150 mL of an aqueous infusion from a 3 g sachet of roselle three times a day, 2 h after a meal. The study lasted four weeks. After the whole period of the intervention, it was shown that the consumption of water infusions of roselle, according to the above-described treatment regimen, significantly lowered the systolic and diastolic blood pressure values in T2DM patients accompanied by mild hypertension, compared to the results before treatment. Contrary to the previously described research, roselle had no effect on the pulse pressure. Also, roselle infusions did not affect the fasting blood glucose level, body weight, or body mass index (BMI) of the participants [178]. A reduction of systolic blood pressure was also demonstrated in patients with diabetic nephropathy by Sakhaei et al. [53]. In this study, patients used pills containing 425 mg of the roselle extracts standardized on anthocyanin content once a day for eight weeks. The results of the study revealed that roselle significantly lowered the systolic blood pressure in patients treated with the pills compared to the group of patients receiving a placebo. What is more, roselle intervention also improved several serological and urine parameters connected with renal function in these patients compared to the placebo-receiving patients or the values recorded before the intervention. However, as in the studies mentioned above, this treatment did not improve the glycemia of the participants [53]. The roselle tea consumed twice a day by older women with metabolic syndrome for 21 days significantly lowered the systolic and diastolic blood pressure in the experimental group compared to the values before the treatment. However, only the systolic blood pressure value after the intervention period was significantly lower than in the control, untreated patients [182]. The ability of roselle to lower systolic blood pressure in adult patients with metabolic syndrome was also demonstrated by Asgary et al. [186]. The patients used one capsule of 500 mg roselle powder standardized on the total anthocyanin content once a day, with food for four weeks without changes in diet and physical activity. The results obtained at the end of the study show that using such a treatment regimen results in lower systolic blood pressure values compared to the values measured at the beginning of the study and when compared to the placebo group [186].

### 5.2. Effect of Hibiscus sabdariffa on the Lipid Profile in Diabetic and Pre-Diabetic Patients

Many clinical trials conducted in diabetic patients treated with roselle revealed that it also reveals a beneficial effect on the lipid profile. For instance, Mozaffari-Khosravi et al. [181] indicated that in patients who consumed 240 mL of tea with a 2 g sachet of roselle two times daily for one month, the HDL level significantly increased, while the level of total cholesterol, LDL, triglycerides, and Apo-B100 in the blood was significantly reduced as compared to their levels before the treatment [181]. Interestingly, in another study Mozaffari-Khosravi et al. [184] showed that roselle only positively influences blood HDL levels. Patients with T2DM consumed 150 mL of infusions with 3 g of roselle three times a day for four weeks. At the end of the study, it was shown that roselle significantly increased blood HDL levels in patients compared with HDL levels at the start of the treatment, but other measured parameters, such as total cholesterol, triglycerides, or LDL levels, remained unchanged, despite the intervention [184]. The effect of roselle on lipid profiles has also been confirmed in patients suffering from metabolic syndrome. In the study, in which the participants were enrolled into three intervention groups (only diet, only HSEP, or HSEP + diet), the treatment with HSEP alone reduced the total cholesterol and LDL levels and increased HDL levels in comparison with the results recorded before the treatment. A combination of diet and HSEP improved the HDL levels, but contrary to the HSEP-only treatment, no effect on the total cholesterol or LDL level was noted. On the other hand, the VLDL and triglyceride levels were reduced after the combined intervention, while HSEP alone did not affect these parameters. Moreover, both types of HSEP interventions improved the ratio of triglycerides to HDL and, interestingly, reduced the blood glucose levels [179]. Similar observations were made in a different study conducted on patients with metabolic syndrome. The patients used one capsule of 500 mg roselle powder standardized on the total anthocyanin content once a day, with food for four weeks. After the treatment period, the HDL level was significantly higher while triglycerides level was significantly lower than at the beginning of the study. Also, the treatment significantly reduced the serum triglyceride levels compared to the placebo group. As far as other parameters connected to the lipid profile were concerned, the intervention did not affect any of them when they were compared either with the baseline values or with the placebo group [186]. Interestingly, Yusni et al. [182], in their studies on older women with metabolic syndrome, showed a negative effect of roselle on HDL levels. Patients were supplemented with 150 mL of tea with 2 g of roselle consumed twice daily for 21 days. After this time, the results showed that roselle significantly lowered the concentration of cholesterol, LDL, triglycerides, and, undesirably, also HDL [182]. In a meta-analysis, Zhang et al. [56] also did not confirm the obvious effect of aqueous roselle extracts on HDL levels in patients with metabolic syndrome. The results of their study confirmed only the reports of their predecessors, that these extracts lower the level of total cholesterol and LDL [56].

The results presented in all aforementioned human studies indicate the therapeutic potential of roselle in diabetic and pre-diabetic patients. Also, a meta-analysis of several randomized clinical trials concerning the efficacy of roselle in treating diabetes-related conditions provided by Bule et al. [171] revealed that this plant possesses antidiabetic properties and can be used in the everyday life of diabetic patients [55]. Obesity is also one of the diseases associated with T2DM. Serna et al. [191] showed in their research that extracts of *Lippia citriodora* and roselle reduce the feeling of appetite, increase satiety, and thus reduce the amount of calories consumed. Therefore, the research results suggest that the consumption of roselle extracts in such a combination may reduce the incidence of obesity and thus reduce the likelihood of T2DM associated with it [191].

## 6. *Hibiscus sabdariffa* as a Prospect in Creating a New Generation of Drugs

The technology of producing nanoparticles and their subsequent use is becoming more and more popular among researchers. There are reports of the use of aqueous or alcoholic roselle leaf extracts in the green synthesis of selenium or zinc nanoparticles [192,193]. The studies showed that such selenium nanoparticles could diminish the effects of damage caused by oxidative stress in the testes of STZ-induced diabetic rats [192]. On the other hand, the abovementioned zinc nanoparticles were able to induce the function of Th1 and Th2 cells as well as increase the expression of insulin receptors and other genes related to diabetes [193]. The results of research on the properties of nanoparticles are very promising and may form the basis for developing technologies for the production of new drugs for diabetes. An interesting fact is that there are studies aimed at finding a way to extend the antidiabetic effect of roselle extracts. One way to do this is to create water-in-oil nanoemulsions. The research results indicate that the kinetics of the release of active substances in the upper gastrointestinal tract at a lower pH is non-linear. It is responsible for the longer residence time of the drug in the gastrointestinal tract, thus, prolonging the antidiabetic effect of roselle extracts. The results of the research provide a promising basis for the development of better preparations of roselle that may complement the pharmacological treatment of diabetes [58].

Moreover, the literature indicates 50 chemical compounds found in roselle with potential hypoglycemic activity and their possible molecular mechanism based on the network pharmacology approach [64]. There are also studies that identify the four main compounds of roselle with great potential for developing anti-diabetic drugs. These are quercetin, hibiscetin, gossypetin, and protocatechuic acid. In in silico studies, they have lower docking scores and higher potential as a phosphoenolpyruvate carboxykinase inhibitors [57]. It provides a good basis for further research into targeted substances and their possible use as antidiabetic drugs [64].

## 7. Conclusions

In this study, we wanted to present the current state of knowledge regarding the impact of *Hibiscus sabdariffa* and its extracts on the course of diabetes and its associated diseases. Evidence of its effects has been sought from in vitro studies, animal experiments, and human trials. The reports that we have collected clearly indicate the multidirectional effect of roselle extracts in the prevention and treatment of diabetes and its accompanying diseases. First of all, roselle and its extracts show a hypoglycemic effect. There is evidence of its ability to increase serum insulin levels (by regenerating the β-cells of the Langerhans islets) and reduce HOMA-IR values in diabetic animals. The strong antioxidant effect of roselle and its protective effect from some diseases have also been reported. The antioxidant properties of this plant improve, among other things, the state of dyslipidemia, which may be of importance, especially in obesity that often coexists with T2DM. The studies showed that roselle has a protective effect on many organs, such as the pancreas, liver, and kidneys, through various mechanisms of action. Roselle and extracts prepared with it can also help to improve cardiovascular dysfunction. Moreover, it protects the testes and semen from damage caused by diabetes. There are also reports indicating that roselle improves cognitive dysfunction in the course of diabetes or intestinal dysbiosis (using roselle as a probiotic). In addition, the antibacterial activity of *Hibiscus sabdariffa* and its potential use as an antiseptic in the diabetic foot has been demonstrated. Roselle also has a hypoglycemic effect in combination with other plant extracts or synthetic drugs. New forms of drugs are also becoming more and more popular, mainly nanoparticles, the production of which uses roselle and which have a high potential for use in the treatment of diabetes. Roselle can be used as a functional food, for instance, as safe drinks for people with diabetes. To sum up, *Hibiscus sabdariffa* is a plant with many medicinal properties and with promising results from in vitro and in vivo research in the treatment or prevention of diabetes and its accompanying diseases (Figure 2). Therefore, it is worth considering its use alone or in combination with convection drugs to counteract the adverse changes caused in the body by diabetes.

## Figures and Tables

**Table 1:** Active chemical compounds in *Hibiscus sabdariffa*.

Chemical Groups	Active Compounds	References
Anthocyanins ^1^	delphinidin-3-sambubioside cyanidin-3-sambubioside	[2,7,18]
Flavonoids ^1^	quercetin, hibiscetin (hibiscetin-3-glucoside), sabdaritrin, gossypitrin, and other gossypetin glycosides, luteolin	[2,7,18,23]
Phenolic acids ^1^	chlorogenic acid, protocatechuic acid, caffeic acid	[2,7,18,23]
Tannins ^1^	no specific name indicated	[18,21]
Non-phenolic organic acids	hibiscus acid, hydroxy citric acid, malic acid, ascorbic acid, oxalic acid, succinic acid, tartaric acid, arachidic acid, citric acid	[2,7,18,22]
Triterpenoids	α-amyrin, lupeol	[24]
Polysaccharides (Sugars)	galactose, galacturonic acid, rhamnose, arabinose, glucose, mannose, xylose, pectins	[7,23]
Others	calcium, magnesium, iron, trace elements, and vitamins	[4,5]

^1^ Polyphenols.

## Data Availability

Not applicable.
